# Echocardiographic Evaluation of Left Atrial Mechanics: Function, History, Novel Techniques, Advantages, and Pitfalls

**DOI:** 10.1155/2015/765921

**Published:** 2015-07-07

**Authors:** Roman Leischik, Henning Littwitz, Birgit Dworrak, Pankaj Garg, Meihua Zhu, David J. Sahn, Marc Horlitz

**Affiliations:** ^1^Faculty of Health, School of Medicine, Witten/Herdecke University, 58448 Witten, Germany; ^2^University of Leeds, Leeds Institute of Cardiovascular and Metabolic Medicine (LICAMM), Leeds LS2 9JT, UK; ^3^Department of Pediatrics, Division of Cardiology, Oregon Health & Science University, Portland, OR 97207, USA

## Abstract

Left atrial (LA) functional analysis has an established role in assessing left ventricular diastolic function. The current standard echocardiographic parameters used to study left ventricular diastolic function include pulsed-wave Doppler mitral inflow analysis, tissue Doppler imaging measurements, and LA dimension estimation. However, the above-mentioned parameters do not directly quantify LA performance. Deformation studies using strain and strain-rate imaging to assess LA function were validated in previous research, but this technique is not currently used in routine clinical practice. This review discusses the history, importance, and pitfalls of strain technology for the analysis of LA mechanics.

## 1. Introduction

The left atrium modulates left ventricular filling and cardiac performance through its roles as a reservoir [1–3], conduit [4], and booster pump [5]. Atrial myocardial deformation properties predict the maintenance of sinus rhythm after external cardioversion [6]. LA function was previously estimated using angiography [3], micromanometry [7, 8], and pulmonary pressure measurements [9, 10]. Doppler techniques [11–14] and strain technology [6, 15–17] are both new methods for the noninvasive evaluation of atrial mechanics [18]. Systemic hypertension patients with contractile function changes, left-sided end-diastolic pressure increases, and volume increases are predisposed to AF (AF). AF is the most common arrhythmia in humans and is characterized by disorganized atrial muscular activation with no effective atrial contraction. The atrial booster pump function is lost due to asynchronous atrial contractions during AF. This loss is associated with a fall in cardiac output, which has particular relevance in ventricular hypertrophy and ischemic heart disease, in which diastolic performance is already abnormal [19]. Standard evaluations of diastolic function using pulsed-wave (PW) Doppler of mitral inflow and tissue Doppler imaging (TDI) supplemented with LA deformation studies can diagnose early LA disease processes, thereby guiding treatments to prevent the development or recurrence of AF. Strain and strain-rate imaging to assess LA function in hypertensive patients with normal LA size demonstrated a lower reservoir function in each LA segment that was independent of age, sex, and heart rate [20].

Patients with diabetes and normal LA size have impaired LA deformation mechanics [21]. Additionally, the coexistence of both diseases impairs LA performance in an additive manner [21].

This paper explains the application of these novel techniques to assess LA function and critically discusses the pitfalls, problems, and impacts of these noninvasive imaging techniques. This review also explains how LA deformation assessments in routine clinical practice may facilitate appropriate AF management strategies and guide treatments to prevent AF.

## 2. History and Invasive Approaches of Atrial Function Assessment

**Figure 1:**
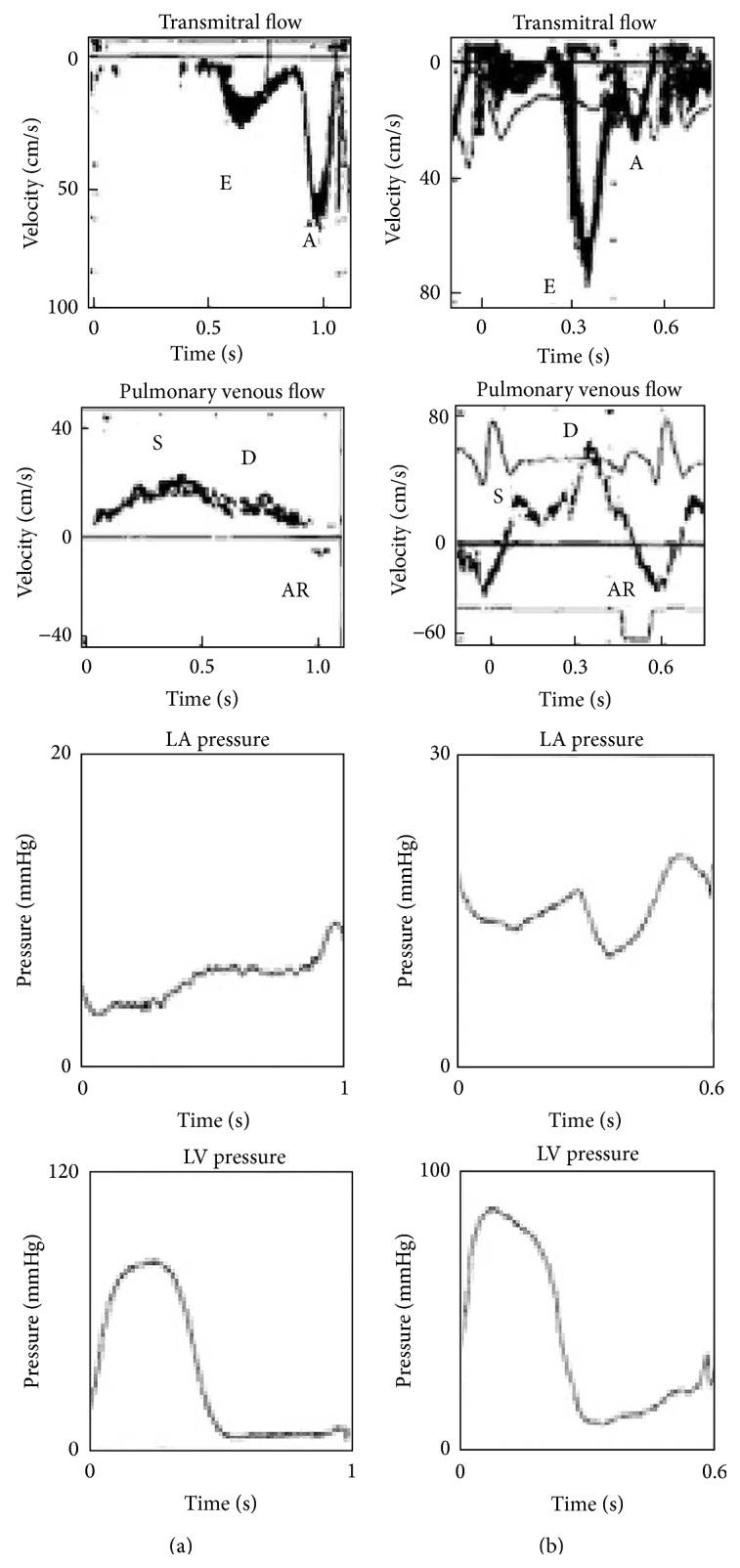
(a) Doppler and hemodynamic flow and pressure curves in patients with a delayed relaxation filling pattern. (b) Patients with restrictive filling patterns, adapted from Thomas et al. [40].

William Harvey discussed the important role of the auricles in 1628:* “blood enters the ventricles… by the beat of the auricles”* and* “… they are filled as reservoirs”* [22]. A young American who studied the physiological properties of frog hearts in Leipzig in 1869 described the special “electric” nature of heart muscle [23]. Contrary to our scientific expectation, Howell and Donaldson but not Starling described the influence of fluid volume on ventricular performance for the first time in 1884. Frank [24] laid the foundation for the basic regulation of the Frank-Starling Law in 1895. Henderson, in 1906 [25], and Henderson and Barringer Jr., in 1913 [26], suggested fixed relaxation and diastolic capacity patterns. In 1911, Gesell [27] described the influence of auricular systole and its relationship to left ventricular output, and Patterson and Starling further described the impact of venous inflow for determining cardiac output and the role of the connections between venous inflow and outflow in the early 1900s [28]. Independently of Starling and coworkers, the German physiologist Straub [29] published the role of diastolic filling (venous pooling) on ventricular performance, and Wiggers [30] examined which factors influenced right ventricular function. Wiggers [31], who directly witnessed the interesting era from 1900 to 1950 subsequently described the determinants of cardiac performance in the 1950s. Braunwald and coworkers initiated research on the invasive measurements of atrial pressure combined with left ventricular pressures [32]. The relationship between increased left ventricular end-diastolic pressure (LVEDP) and elevated mean LA pressure in patients with left ventricular disease was described for the first time in 1961 [33]. The conduit function can be measured invasively [34] using volume/pressure curves. The experimental and hemodynamic era of heart examinations laid the groundwork for all future noninvasive investigations. Suga [4] concluded that atrial compliance was an important determinant of heart performance as a whole. The contractile nature of nature atria is not fully consistent with the present assumptions that atrial compliance is linear and constant [4]. The normal filling process of more than half of the ventricle during diastole before atrial contraction must be considered. Atrial contraction and deformation are consecutively affected in pathological situations in which the elastic properties of the myocardium are altered [35], leading to changes in the secretory function of the atria [36]. The hemodynamic role of the atria in regulating sodium and as a secretory organ dominated the literature in the late 1980s [36, 37]. The elastic properties of the myocardium and diastolic function have many determinants, which represent a broad area of research [7]. Ideally, simultaneously conducted chamber pressure, volume, flow, and neurohormonal factor (e.g., adrenalin, cortisol, and atrial natriuretic factor) measurements should be considered to examine the LV and LA elastic properties and systolic functions. In 1986, Ishida et al. [38] documented changes in transmitral flow, especially reduced early diastolic transmitral flow during increased afterload, using an invasive technique. Left ventricular filling dynamics and the influence of ventricular relaxation on LA pressure were examined in dogs and were compared to mitral flow. In 1988, Appleton et al. showed a significant relationship between pulmonary wedge pressures and transmitral Doppler velocities [11]. In 1991 [39] and 1997 [40], Thomas et al. compared LA pressures, left ventricular pressures, and transmitral Doppler flow or pulmonary venous flow (Figure 1). Ommen et al. [41] and the Mayo clinic working group simultaneously documented the clinical utility of Doppler and TDI in a comparative Doppler-catheterization study and demonstrated that the noninvasive assessment of LV filling pressures is an important clinical tool.

## 3. 2D/3D Echocardiography 

Echocardiography provides a broad range of information on anatomic and functional changes to heart structures over time [42, 43]. Two-dimensional/three-dimensional (2D/3D) echocardiographic evaluation offers robust structural and functional information for the entire heart. Time-tested one-dimensional M-mode echocardiographic recordings can examine changes over the systolic and diastolic time periods during the cardiac cycle [44]. LA size is commonly estimated using M-mode-derived diameters. Reliable measurements can only be obtained using multiplane measurements [45, 46]. LA size is a strong predictor of clinical outcome in several conditions [47–49]. LA size has been proposed as a barometer of diastolic burden and an indicator of the magnitude and duration of diastolic disease [50]. The measurement of LA size in M-mode must be avoided due to the ovoid shape of the atrium. However, further evaluations of LA reservoir function and LA stiffness using 2D measurements have been validated [2]. 3D echocardiography was recently used to reliably measure LA sizes/volumes [51], and this method is the most robust solution for reliable LA size measurements [52, 53]. 3D echocardiography yields more reliable size/volume measurements but is more time consuming; furthermore, the differences in LA volume demonstrate only a minor, nonsignificant improvement [54]. The combined use of conventional 3D echocardiography and strain technology to assess LA size and function will likely play a more definitive role in the future [55, 56]. A central role for the assessment of atrial function using this technology is discussed further in this paper.

Using conventional 2D echocardiography, Saraiva et al. [16] obtained practicable measurements and normal values (Table 1(a)), and Todaro et al. [18] obtained complementary measurements (Table 1(b)).

## 4. PW-Doppler, PW-TDI, and Color TDI

Diastolic disease was already clinically recognized in the early 1980s [12, 57, 58] but was difficult to detect or quantify by all except well-educated cardiologists until 1982 [14]. Before the introduction of pulsed Doppler by Kitabatake et al. in 1982 [14], echocardiography using mitral leaflet motion analysis was used to demonstrate impaired early ventricular filling [59]. The assessment of diastolic heart failure using Doppler has become a fixed, integral part of European working group guidelines [60, 61]. PW-Doppler, which was validated using invasive measurements [11, 40, 41, 62], is the most widely used technique to directly analyze diastolic function and indirectly analyze atrial function [2, 11, 12, 14, 35, 63]. Mitral and pulmonary flow velocities reliably correlate to mean LA pressures and to pressure changes [39, 40, 62]. LA function has 3 phases: reservoir (inflow during ventricular systole), conduit (passive emptying during ventricular relaxation and diastasis), and contraction (active emptying near ventricular end-diastole) [2]. All of these properties are easily analyzed using PW-Doppler [64, 65]. Impaired diastolic function can be detected using a simple method and elementary echocardiography equipment [35, 66, 67]. The simplicity of this technique is its most important advantage (Figure 1) based on all known reservations today [68].

A lower peak early diastolic velocity (*V*_max⁡_ E = passive inflow) and a comparatively higher atrial contraction velocity (*V*_max⁡_ A = active inflow) are established signs of diastolic dysfunction [64, 66]. The combined use of Doppler measurements and 2D echocardiography was proposed to estimate the “atrial ejection force (AEF)” (Mass × Acceleration) [69]. In this case, the following formula was used:

(1) Atrial  ejection  force=0.5×p×Mitral  orifice  area×Peak  A  velocity2,

where *p* is the density of blood (*p* = 1.06 g/cm^3^) and the units of force are measured in g-cm/s^−2^ or dynes.

LV diastolic dysfunction measured using a simple Doppler technique is a strong predictor of first-diagnosed nonvalvular or incident AF [48, 70]. Diastolic disease is the leading cause of the atrial enlargement and LA pressure elevation associated with AF [65]. Sutherland et al. (1994) presented the first clinical use of color TDI [71], and Donovan et al. used this technique in healthy volunteers [72]. Sohn et al. [73] first described a “pseudonormalization” of diastolic function using Doppler indices (PW-TDI) by comparing transmitral PW-Doppler velocities to PW-TDI recordings on the septal side of the mitral annulus. Pseudonormalization was described as a normal pattern of transmitral flow (E > A) with reverse velocities derived by PW-TDI (E′ < A′). Sohn et al. presented a connection to the suggestions of Sutherland et al. [71] based on suggestions of Alam and Hoglund [74] on examinations of diastolic atrioventricular plane displacement using M-mode echocardiography. Within the same year, Nagueh et al. [75] published similar observations using PW-TDI measurements from the septal and lateral sides of the mitral annulus. PW-TDI measurements of the lateral side of the mitral annulus showed higher velocities than those of the septal side. Park et al. suggested that the lateral E/Ea PW-TDI ratio may be more reflective of true LV diastolic function [76]. Similar observations were described by Kim et al. [77] using TDI. This working group suggested an overestimation of diastolic dysfunction using septal e′/a′ and proposed a lateral TDI e′/a′ ratio as an indicator of early but not of advanced diastolic dysfunction in subjects with a PW-Doppler mitral inflow ratio of E/A > 1 and a septal TDI ratio of e′/a′ < 1.

The diastolic motion of the mitral annulus measured by PW-TDI was validated in animal experiments using invasive measurements [78]. Garcia et al. suggested new Doppler echocardiographic applications for evaluating diastolic function [79]. Color TDI was suggested for analyzing diastolic function, but the introduction of this technique into routine clinical use failed.

Poulsen suggested 4 grades of diastolic dysfunction in his doctoral thesis [12]. Based on Sohn et al. [73] and his own serial investigations [63, 80], Poulsen suggested 4 grades of diastolic dysfunction in his doctoral thesis [12].

**Figure 2:**
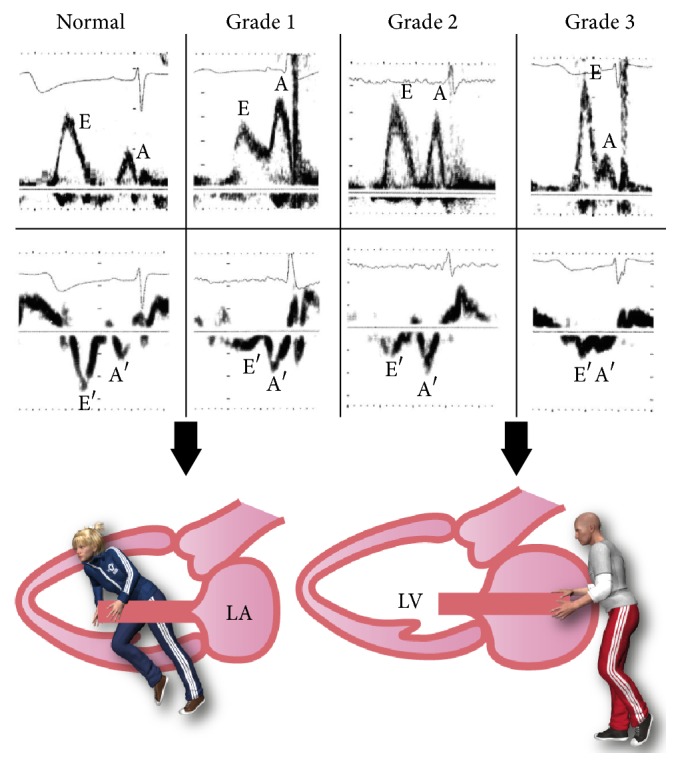
Grade one shows a reduced E/A ratio mitral inflow, measured by PW-Doppler, and a reduced E′/A′ ratio, measured by PW-TDI. Grade 2 represents “pseudonormalization,” and Grade 3 represents restrictive physiology [35, 73].

Oh et al. [35] postulated 3 grades of diastolic dysfunction (Figure 2). These authors illustrated a schematic diagram of the “sucking” of blood into the left ventricle from the left atrium by good relaxation or the “pushing” of blood into the left ventricle by an increased filling pressure in patients with diastolic dysfunction.

**Figure 3:**
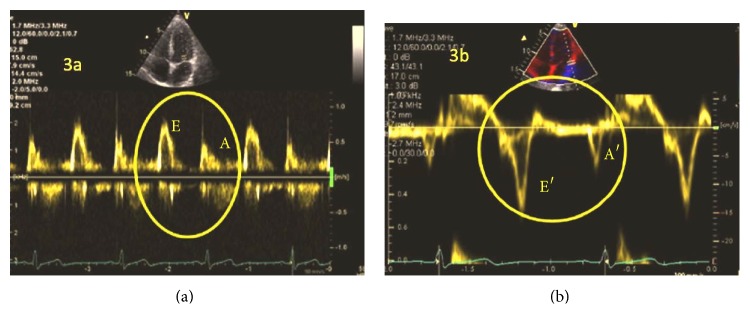
Normal diastolic function in PW-Doppler (a) or TDI (b) on the lateral side of the mitral annulus.

Of all of the proposed parameters, only e′ (E′) and a′ (A′) derived from TDI (Figure 3(b)) (earlier from conventional PW-Doppler) [41] were validated and passed into routine clinical practice. The parameters were used with varying relevance [35, 68, 77] and in different combinations [68] and were associated with the pitfalls of angle dependency [71, 81], reproducibility, and invasive validation [68, 82] (Figure 3(b)). Hayashi et al. [81] demonstrated angle dependency and lower mitral annulus motion measurement values using TDI.

Kasner et al. [68] did not recommend the single use of PW-Doppler mitral inflow measurements and proposed the LV filling index E/E′_lateral_ as the best index to detect diastolic dysfunction in patients with heart failure and a normal ejection fraction (EF); Ommen et al. [41] suggested a similar index.

Mullens et al. [83] suggested that the E/E′ ratio (E/Ea ratio in their publication) is not reliable in patients with advanced systolic heart failure. Thus, the E/E′ ratio alone may not be reliable in predicting intracardiac filling pressures. The authors suggested a need to refine the broad clinical use of the mitral E/E′ ratio to estimate filling pressures and cautioned against the direct inference of relationships in patients with a decompensated state with significant LV systolic dysfunction, cardiac remodeling, or biventricular pacing.

Galderisi et al. [84] recommended using the lateral E/E′ ratio to predict a pulmonary capillary wedge pressure (PCWP) >18 mmHg in patients with coronary disease.

Bhella et al. [85] made a radical statement regarding echocardiographic indices derived from TDI ratios. The authors advised that the noninvasive indices E/E′ and E/Vp are not reliable for tracking changes in the left-sided filling pressure in healthy subjects or in patients with heart failure and preserved EF.

**Figure 4:**
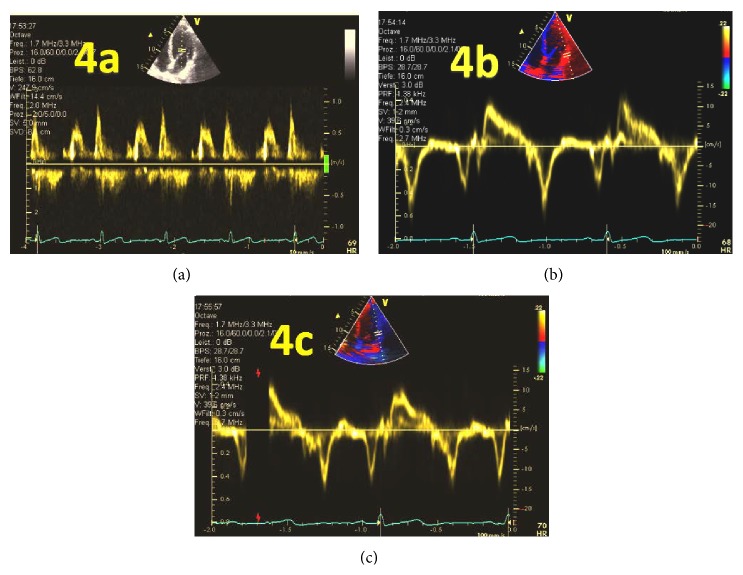
Pitfalls of the E/A and E/E′ ratio measured using PW-Doppler and TDI. Different values for diastolic dysfunction when measurements are performed using (a) PW-Doppler; (b) TDI measurement of lateral mitral valve annulus motion; (c) TDI measurement moved light to the lateral LV wall.

Despite the reliability of TDI [82], problems with the measurements include day-to-day variability [86] and inter- and intraobserver reproducibility [82, 86]. Additionally, dislocations from the mitral annulus to the lateral ventricular wall may produce different measurements (Figures 4(b) and 4(c)). According to statistical decision and information theory principles for decision making, using the recommended indices for decision making becomes problematic due to the many different opinions regarding TDI-derived parameters [87].

As a whole, the heart acts as a suction pump, but the atrium represents an important component of a functioning cardiovascular system [88]. The muscular architecture of the atrium [16, 89] and the left ventricular fibers [90] reveal the impact of deformation on atrial [16, 89, 91] and ventricular function [92–94]. TDI [95] and strain imaging [96] were initially used as a diagnostic tool to detect ischemia but were further used [15] to identify strong indicators of diastolic disease-associated diastolic dysfunction and atrial burden.

## 5. Strain

Di Salvo et al. [6] measured atrial wall velocities using sample volumes in patients with AF and reference subjects and calculated strain/strain rate values of the atrial walls. AF patients exhibited lower velocities using color TDI; however, interobserver values were not investigated in this study. Di Salvo et al. suggested peak systolic and peak early diastolic values of atrial velocity measured using TDI and peak systolic and peak early diastolic values measured using strain and strain rate, but only segmental values were examined. The estimated differences were significant between the two groups, but the differences were not sufficiently significant for use as a relevant specific diagnostic tool in the future. Based on a publication by Greenbaum et al. [90] and technical development, the deformation analysis ideas were introduced into the cardiology circuit [15, 94, 97].

**Figure 5:**
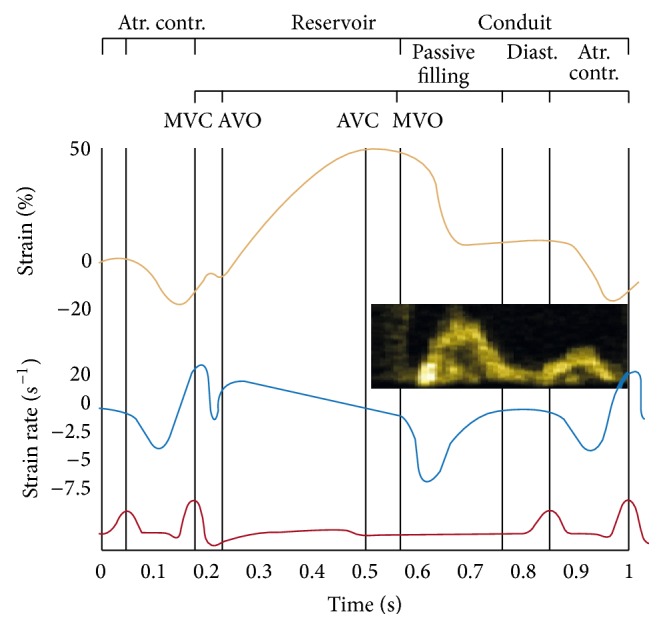
Atr. Contr: atrial contraction/contractile function of left atrium, MVC: mitral valve closure, AVO: aortic valve opening, AVC: aortic valve closure, MVO: mitral valve opening, and Diast.: diastasis.

Sutherland et al. first validated the clinical use of TDI to assess left ventricular function in 1994 [71]. His group (Sirbu et al.) subsequently demonstrated the value of LA strain imaging in assessing LA function [98]. This study first suggested using strain technology to analyze global atrial function and proposed 3 points on the strain (*ε*) curve as indicators of LA function: contractile function from 0.1 to 0.2 seconds, reservoir function from 0.3 to 0.5 seconds, and conduit function from 0.5 to 0.7 seconds (Figure 5).

The possibility for different estimations of “peak” values or time periods using strain (*ε*) or strain-rate curves exists during the time intervals of ECG intervals. Whether septal atrial wall or only “outside” mobile walls (lateral/posterior/anterior) should be considered remains unclear. Therefore, the possibility of introducing error exists using the segmental strain of the LA to estimate global strain values. Kokubu et al. [99] measured segmental atrial strain rate values in two-, three-, and four-chamber views by TDI in hypertensive patients after treatment with renin-angiotensin system (RAS) inhibitors. They proposed that strain rate imaging could detect LA dysfunction. A small significant difference was observed between hypertensive patients with and without dilated LA. An interobserver study was not performed, and the differences were not clinically useful.

Thomas et al. [100] proposed an atrial strain rate derived from a point on the midatrial septum as a marker for dysfunction; however, similarly to previous studies, this parameter did not initially merit much research or clinical impact. The idea of using and optimizing cardiac mechanics to assess LA function was eventually revisited in 2008 [101]. Schneider et al. suggested using mean strain and strain rate values measured during systole (LAs) and at early (LAe) and late (LAa) diastole as indicators of reverse atrial remodeling. Patients with higher atrial strain and strain rates after catheter ablation appeared to have a greater likelihood of maintaining sinus rhythm. The differences were small but significant and were derived from clinically useful values. However, a cut-off value could not be estimated. Cameli et al. [102] proposed global LA longitudinal strain values derived by speckle tracking of the left atrium, and peak atrial longitudinal strain (PALS) and time to peak longitudinal strain (TPLS) measurements were also proposed.

Kim et al. [91] proposed the use of global LA longitudinal strain during systole and early and late diastole, as well as of the related peak strain values. They showed no evidence of any systematic difference in the intra- or interobserver variability in 10 patients from their 54-subject study population. Vianna-Pinton et al. [103] demonstrated that the 2D speckle tracking technique was angle independent and was feasible in 94% of normal patients to assess regional differences in LA contractility using regional LA strain (LA*ε*) and the regional LA strain rate (LA*ε*′). The interobserver variability of global values was 5.7% for velocity and 6.5% for strain. A total of 13 LA regions, 5 regions adjoining the mitral annulus, and 5 regions in the midatrial wall were examined; in each view, the superior, or “roof,” region, corresponding to the area encompassing the region between the 4 pulmonary veins, was also examined [103]. This paper described a specific time schedule of atrial contraction (early atrial activation of midseptal annulus and roof and later activation of the anterior and lateral annulus were suggested).

Saraiva et al. [16] suggested the use of LA strain measured using 2D speckle tracking as a new tool to evaluate LA function. They transcribed a similar methodology as the Sutherland working group: the *ε* positive peak, *ε* negative peak, and *ε* total (*ε* = strain (%)) were the suggested values. This working group suggested the following strain rate values: late negative peak strain rate (SR_late  neg  peak_), positive peak strain rate (SR_pos  peak_), and early negative peak strain rate (SR_early  neg  peak_). The global LA negative strain (LA*ε*) and global LA SR late negative peak were not correlated with the active LA stroke volume or active LA emptying function [16].

Additionally, an assessment of the electromechanical delay (ca. 60 ms) between the interatrial septum and lateral wall was proposed [18].

Additional research groups [18, 104–106] suggested strain imaging as a novel echocardiographic technique to assess LA function. Cameli et al. [104] described systolic strain as peak atrial longitudinal strain (PALS) and atrial contraction as peak atrial contraction strain (PACS). Subsequently, 3D echocardiography was also suggested as a novel technique using strain analysis to assess LA function.

Speckle tracking was used to evaluate atrial function in different patient groups, including those with hypertension [20, 21, 107], diabetes [21, 107], systemic sclerosis [108], and hypertrophic cardiomyopathy [109], as well as in healthy athletes [109, 110]. Mondillo et al. [21] demonstrated impaired atrial strain values in hypertensive or diabetic patients. The coexistence of both conditions further impaired LA performance. Recently, Sahebjam et al. [20] demonstrated reduced strain and strain rate values in hypertensive patients compared with healthy controls.

Schneider et al. suggested that atrial strain and strain rate values of the atrial septum might predict sinus rhythm maintenance after AF ablation. In 2014, Spethmann et al. showed similar results in their study [111]. They suggested that LA mechanics might predict the success of pulmonary vein isolation in patients with AF. Peak positive strain (in this study, reservoir function = R_LA_), early diastolic strain (conduit function = E_LA_), and active atrial contraction (A_LA_) were used in a pilot study prior to pulmonary vein ablation. The described parameters exhibited sufficient intraobserver variability (0.97 for R_LA_, 0.92 for E_LA_, and 0.99 for A_LA_).

Severe mitral regurgitation is associated with LA dysfunction related to the presence of indications for mitral surgery. LA dysfunction measured using reservoir strain may be a valuable clinical marker for follow-up and decision making in conventional mitral surgery [112]. The reservoir function (strain, in %) and active emptying fraction (%) were significantly reduced (19 ± 7.7 versus 31 ± 6.1 and 24 ± 10 versus 32 ± 12, *P* = 0.001) in patients with mitral regurgitation and indications for mitral surgery compared with controls [112].

Diminished augmentation of the LA reservoir and passive emptying functions during dobutamine stress were strongly associated with cardiovascular events [113]. Assessment of the LA functional reserve may result in further improvements in prognostic risk stratification in patients with dilated cardiomyopathy (DCM) [113].

Hammerstingl et al. described reduced global longitudinal atrial strain as a predictor of AF recurrence [114]. In a 4-chamber view, global LA strain was significantly reduced in patients with recurrent AF compared with sinus rhythm maintenance (5.1 ± 6.7 versus 22.9 ± 11.7, *P* = 0.0005).

## 6. Conclusions 

**Figure 6:**
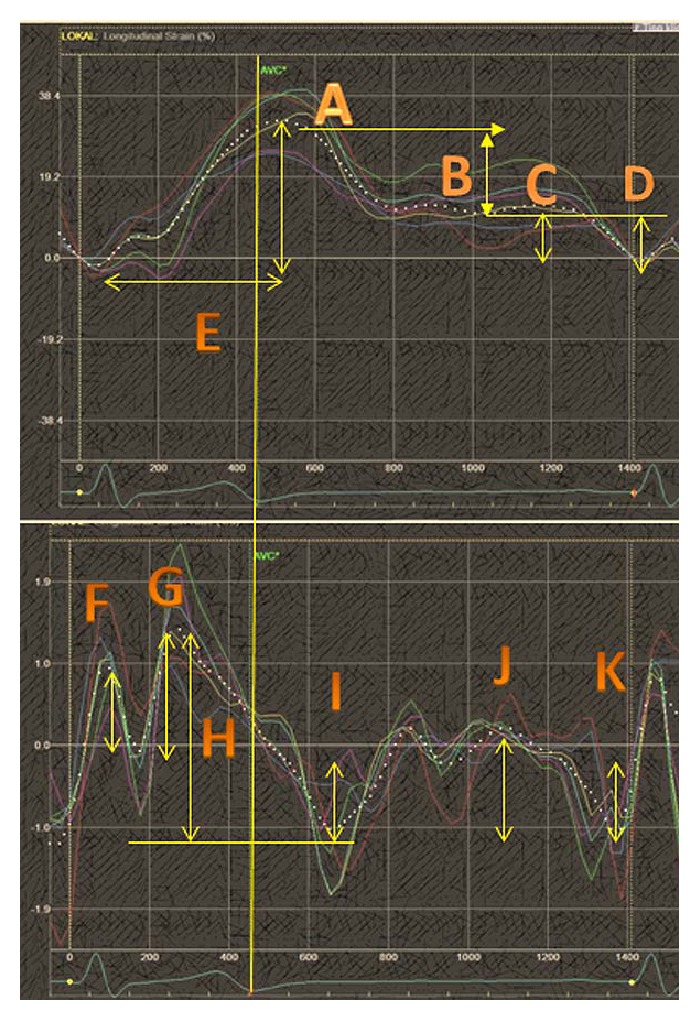
Suggested strain values; A: peak systolic strain (reservoir function), B: peak early diastolic strain (conduit function), C: peak late diastolic strain, D: peak negative diastolic strain, E: time to peak systolic strain, F: early peak systolic strain rate, G: late peak systolic strain rate, H: total systolic strain rate, I: peak negative early diastolic strain rate, and J: peak positive late diastolic strain rate.

Strain imaging [115] is the most promising technology for the direct evaluation of LA function [116]. This imaging technique offers many opportunities to measure several quantitative parameters but unfortunately lacks clear standards and validation (Figure 6). Strain imaging begins with the establishment of the onset of time-point markers for analysis (QRS, atrial wave, or aortic closure) and ends with standardized online/offline atrial analyzing software tools from different vendors, which are lacking.

**Figure 7:**
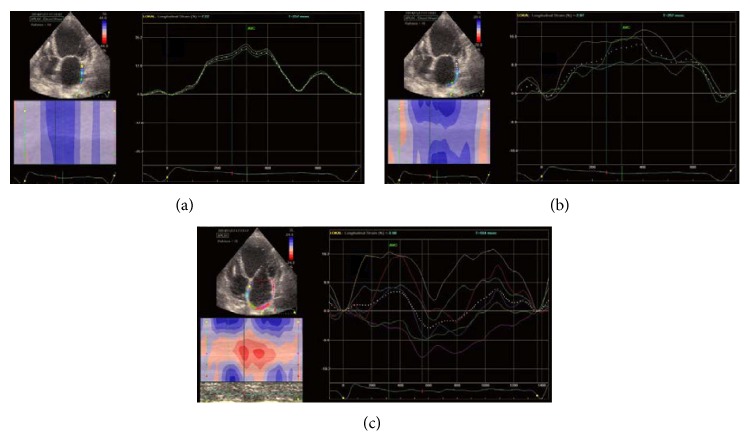
(a) Three-segment small extension (only the lateral wall), (b) three-segment larger extension (lateral and posterior walls), and (c) all six segments; different time points give different values.

All current software options were designed to conduct left ventricle analyses; less software is available for atrial strain analyses. There is also a lack of standards or official consensus documents. The underlying problems are the theoretical presence of three segments, at a minimum, which can be placed in different positions and extensions or the analysis of six segments in different echo views (Figure 7). Different extensions and different time points lead to different values.

The four-chamber view (the most commonly used) provides atrial septal movement, which may influence the analyses as a whole in an unclear manner. Other issues include the relatively small “cut-off” values or overlapping areas for differential diagnoses or disease stages [87]. We have not progressed much further regarding establishing standards and reducing pitfalls between the first inception of strain imaging for the analysis of LA function [98] and the latest review [116]. The old parameters (Tables 1(a) and 1(b)) are valid, and the LA EF has a similar potential as strain to identify recurrent AF [114]. Doppler parameters such as the E/A ratio are valuable and easily obtained, and, most importantly, these parameters have stood the test of time. Global LA strain in the four-chamber view likely offers three interesting parameters in the absence of segmental failures of deformation: maximum positive strain (reservoir Figure 6(A)), late atrial positive strain (Figure 6(C)), and peak negative diastolic strain (Figure 6(D)). These two LA strain imaging parameters (Figures 6(A) and 6(B)) seem to have the most evidence [104] to support their clinical role. All strain parameters (Figure 6) required further standardization and additional comprehensive studies.

The development of a standardized software tool for the specific analysis of LA function and consequent experimental studies using the standardized parameters (either global or segmental) are needed. The measurement onset and time points must be standardized (onset QRS, P-wave, and aortic closure). Evaluations of intra- and interobserver variability according to the ventricular strain are required for all parameters [117]. The atrial wall is thinner, with less muscular mass; therefore, deformation measurements might display more variability on different ultrasound systems. Systematic studies are required in this field to develop clear, clinically valuable standards.

## Figures and Tables

**(a):** 

Variable	Value
Maximum LA volume index (mL/m²)	21.9 ± 5.1
Minimum LA volume index (mL/m²)	7.3 ± 5.0
Precontraction LA volume index (mL/m²)	12.1 ± 4.4
Total LA stroke volume (mL)	28.0 ± 7.7
Total LA emptying fraction (%)	70.3 ± 9.2
Active LA stroke volume (mL)	10.6 ± 5.0
Active LA emptying fraction (%)	46.6 ± 11.7
Passive LA stroke volume (mL)	17.5 ± 6.0
Passive LA emptying fraction (%)	44.3 ± 12.1
LA expansion index (%)	271.5 ± 126.4

Total LA stroke volume: maximum LA volume − minimum LA volume. Active LA stroke volume: precontraction LA volume − minimum LA volume. Passive LA stroke volume: maximum LA volume − precontraction LA volume. The total LA emptying fraction: (total LA stroke volume/maximum LA volume) × 100. The active LA emptying fraction: (active LA stroke volume/precontraction LA volume) × 100. The passive LA emptying fraction: (passive LA stroke volume/maximum LA volume) × 100. LA expansion index: (total LA stroke volume/minimum LA volume) × 100.

**(b):** 

LA passive volumes	
(i) Preatrial contraction volume (*V*_preA_), measured at the onset of the P-wave using an electrocardiogram (ECG).	
(ii) Minimal LA volume (*V*_min⁡_), measured at the closure of the mitral valve and end-diastole.	
(iii) Maximal LA volume (*V*_max⁡_), measured just before the opening of the mitral valve in end-systole.	

LA active volumes	
(i) LA reservoir volume (*V*_max⁡_ − *V*_min⁡_)	
(ii) LA conduit volume (LV total stroke volume − LA reservoir volume)	
(iii) LA passive emptying volume (*V*_max⁡_ − *V*_preA_)	
(iv) LA contractile volume (*V*_preA_ − *V*_min⁡_)	
